# Treatment strategies with combined agency against severe viral pneumonia in patients with advanced cancer

**DOI:** 10.2478/jtim-2024-0007

**Published:** 2024-07-27

**Authors:** Fan Zhang, Tao Li, Yibing Bai, Jiayi Liu, Jiapei Qin, An Wang, Yimin Zhu, Min Zhang, Zhiqiang Ma, Xin Zhou, Lijie Wang, Ming Gao, Xiaodong Wu, Yan Shao, Xiangfei Zhao, Juyi Wen, Jingzhi Guan, Jinliang Wang, Junxun Ma, Haitao Tao, Yi Hu

**Affiliations:** Senior Department of Oncology, The Fifth Medical Center of PLA General Hospital, Beijing 100039, China; Medical School of Chinese PLA, Beijing 100853, China; School of medicine, Nankai University, Tianjin 300071, China; Department of Oncology, The Eighth Medical Center of PLA General Hospital, Beijing 100091, China; Senior Department of Nephrology, The First Medical Center of PLA General Hospital, Beijing 100853, China; Department of Oncology, the Sixth Medical Center of PLA General Hospital, Beijing 100048, China

## To the editor

Patients with cancer are particularly vulnerable to severe acute respiratory syndrome coronavirus 2 (SARS-CoV-2) infection and experience worse outcomes compared with those without cancer.^[1]^ The 30-day mortality rate associated with SARS-CoV-2 ranges from 13% to 33% in patients with cancer compared with 0.5% to 2% in the general population.^[2,3]^ Immune homeostasis is impaired in cancer patients due to the disease itself, associated comorbidities, or anti-cancer treatments, making them more susceptible to SARS-CoV-2 infection. This emphasizes the critical need for effective and safe treatment strategies to manage SARS-CoV-2 infection in this patient population.^[4]^ Antiviral treatment combined with immunosuppressive agents is recommended as an important part of therapy for patients with severe SARS-CoV-2 infection. However, there remains an unmet need for treatment strategies in patients with advanced cancer and severe SARS-CoV-2 infection.

This study was a multicenter, retrospective investigation conducted between October 1, 2022 and February 1, 2023 at four hospitals designated in patients with cancer and SARS-CoV-2 infection in Beijing, China, including the First Medical Center, the Fifth Medical Center, the Sixth Medical Center, and the Eighth Medical Center of the People’s Liberation Army (PLA) General Hospital. Patients with advanced cancer and SARS-CoV-2 infection were treated with supportive care alone, the PROMISING regimen, or other regimens that included 2–5 medications. The PROMISING regimen comprised supportive care, glucocorticoids, antiviral agents such as Paxlovid, antibiotics for the prevention and treatment of bacterial or fungal infections, human intravenous immunoglobulin (IVIG), and tocilizumab. To analyze the efficacy of different regimens, propensity-score matching was used to create case and control groups with similar baseline characteristics. Matching at a ratio of 1:3 was conducted, with propensity scores estimated based on age, sex, the Eastern Cooperative Oncology Group (ECOG), smoking history, body mass index (BMI), and tumor types. Finally, seven patients who received the PROMISING regimen and 21 patients who received other treatments were included in the analysis.

The primary endpoint of this study was a 28-day SARS-CoV-2-related mortality rate (defined as death related to SARS-CoV-2 infection from patient admission to 28-day follow-up). The secondary endpoints included the time to alleviation of SARS-CoV-2-related symptoms (time for oxygen saturation to improve and return to the normal range or to achieve improvements in patient-reported symptoms), changes in laboratory indicators (time for leukocyte and neutrophil counts in a routine blood test to return to the normal range and to achieve a decline in inflammatory mediators, such as C-reactive protein (CRP), interleukin-6 (IL-6), or procalcitonin), and imaging findings (time to remission according to the imaging grading system), all stratified by treatment regimen. In addition, the safety of the treatment regimens was assessed.

A total of 89 hospitalized patients (69 males and 20 females) with advanced cancer and SARS-CoV-2 infection were included. The median age of the patients was 62 (31–91) years. Among them, 56 patients had severe or critical SARS-CoV-2 infection. Seventeen (19.1%) patients had received at least one type of anticancer treatment within 14 days of SARS-CoV-2 diagnosis. Comorbid lung diseases included but were not limited to chronic obstructive pulmonary disease (COPD), emphysema, and pulmonary heart disease. The most common symptoms upon hospital admission were fever (64.0%), cough (55.1%), and chest tightness (53.9%). The detailed information is shown in Table S1.

The 28-day SARS-CoV-2-related mortality in patients with advanced cancer and severe SARS-CoV-2 infection was 30.4%. Of the 56 patients with advanced cancer and severe SARS-CoV-2 infection, seven were treated with the PROMISING regimen, and all recovered (100%). Among the 49 patients who received other regimens, 3, 6, 19, and 21 received a 5, 4, 3, or 2-medication combination regimen, with mortality rates of 33.3%, 33.3%, 31.6%, and 38.1%, respectively. The 28-day SARS-CoV-2-related mortality among patients who received other regimens was 34.7% (17/49, *P* = 0.049, Table S2), with 32 patients recovering and being discharged and 17 patients succumbing to the infection.

Table 1 shows the logistic regression analysis identifying the risk factors for severe SARS-CoV-2 infection in patients with advanced cancer. The univariable analysis revealed that age (OR 3.556, 95% CI, 1.401–9.022, *P* = 0.008), ECOG performance score (OR 5.214, 95% CI, 1.759–15.451, *P* = 0.003), smoking history (OR 2.870, 95% CI, 1.168–7.047, *P* = 0.021), and comorbid lung disease (OR 8.763, 95% CI, 3.087–24.875, *P* < 0.0001) were risk factors for severe SARS-CoV-2 infection in patients with advanced cancer. The multivariable analysis confirmed that age (≥ 63), ECOG performance score 3–4, and comorbid lung disease were independent risk factors for severe SARS-CoV-2 infection in these patients.

**Table 1:** Logistic regression model of riskfactorsfor severeSARS-CoV-2infectionin patientswith advanced cancer

Variables	Univariable logistic regression 95% CI			Multivariable logistic regression		
	OR	95% CI	*P* value	OR	95% CI	*P* value
Gender (female/male)	2.870	1.168–7.047	0.210			
Age (**≥**63 years/<63 years)	3.556	1.401–9.022	0.008	5.988	1.496–23.976	0.011
Smoking history (Smoker/Never smoked)	2.870	1.168–7.047	0.021			
BMI (**≤**25/**>**25)	1.200	0.486–2.962	0.693			
ECOG(3–4/0–2)	5.214	1.759–15.451	0.003	5.611	1.266–24.858	0.023
Tumor types (lung cancer/other)	1.500	0.612–3.677	0.375			
Comorbid lung disease (YES/NO)	8.763	3.087–24.875	< 0.0001	18.456	3.999–85.167	<0.0001
Last treatment for cancer <14 d (YES/NO)	0.444	0.152–1.297	0.138			
WBC (>10 or <3.5/3.5–10)	1.056	0.383–2.911	0.917			
IL-6 (>5.9 pg/mL/**≤**5.9 Pg/mL)	0.035	1.136–36.568	6.444			
CRP (>0.8 mg/L/**≤**0.8 mg/L)	24.667	1.703–357.361	0.019			
LDH (>250/**≤**250)	2.258	0.655–7.786	0.197			

BMI: Body Mass Index; Eastern Cooperative Oncology Group (ECOG) Performance Status; IL-6: Interleukin-6; CRP: C-reactive protein; LDH: Lacticdehydrogenase; PLT: platelet.

**Figure 1:**
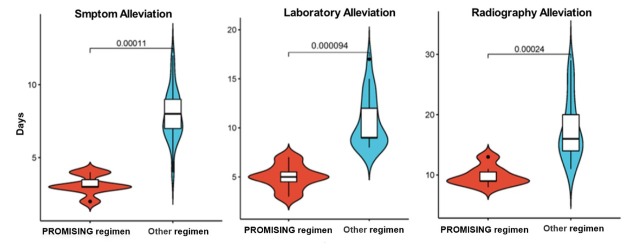
Violin plot of time to *alleviation* of SARS-CoV-2-related symptoms in patients with advanced cancer treated with the PROMISING regimen or other treatment regimens.

The demographic and baseline clinical characteristics of different treatments in patients with advanced cancer were similar between different groups (Table S3). The median length of hospital stay (8.14 d *vs*. 16.86 d, *P* = 0.03), median time to alleviation of SARS-CoV-2-related symptoms (3.14 d, *vs*. 7.95 d, *P* < 0.01), and change in laboratory indicators (5.00 d *vs*. 11.48 d, *P* < 0.01) and imaging findings (9.14 d *vs*. 17.62 d, *P* < 0.01) were significantly shorter in patients with advanced cancer and severe SARS-CoV-2 infection treated with the PROMISING regimen compared with the other regimens (Figure 1). The most frequent adverse events linked with the PROMISING regimen included hyperglycemia and liver damage. The most frequent adverse events associated with the other regimens were secondary infection, hyperglycemia, and gastrointestinal reactions. All adverse events were resolved with symptomatic treatment (Table S4).

This retrospective study investigated the clinical characteristics and compared treatment strategies in patients with advanced cancer and SARS-CoV-2 infection. All patients had advanced cancer, had received multiple lines of anti-cancer treatments, and had poor health status and immune function at admission. These patients were at high risk of progressing to severe SARS-CoV-2 infection and developing severe anoxia. The SARS-CoV-2 infection rate and SARS-CoV-2-related mortality were high. Older age, poor ECOG scores, and the presence of comorbid lung disease were risk factors for severe SARS-CoV-2 infection in our patients.^[5]^ Consistent with this, previous evidence suggests that older age and poor overall health status are predictive of functional decline of multiple organs in patients with advanced cancer.^[6]^

In this study, the PROMISING regimen was associated with decreased SARS-CoV-2-related mortality compared with the other regimens and was linked to a shortened length of hospital stay, time to alleviation of SARS-CoV-2-related symptoms, and changes in laboratory indicators and imaging findings. These data imply that the PROMISING regimen exerts a synergistic effect by providing immune protection and improving overall patient health status, representing an effective treatment option for high-risk patients.

The PROMISING regimen includes supportive care, glucocorticoids, Paxlovid, tocilizumab, gamma globulin, and antibiotics. The development of this regimen was guided by evidence-based medicine and basic science. Steroid administration has shown clinical benefit among all severity categories of patients with SARS-CoV-2 infection in previous meta-analyses and several studies.^[4]^ Paxlovid was reported to be highly effective in reducing the risk of severe SARS-CoV-2 infection or mortality in a population-based, real-world study of adults with a first-ever positive test for SARS-CoV-2 in 2022 during the omicron era.^[7]^ Paxlovid can quickly reduce viral load, aiding virus clearance by the immune system and alleviating symptoms. Early administration of paxlovid may prevent excessive immune response and decrease the long-term persistence of SARS-CoV-2 or its remnants in the body. Tocilizumab may be beneficial for patients with SARS-CoV-2 infection as elevated cytokine levels, particularly IL-6, are associated with severe SARS-CoV-2 infection.^[8,9]^ In a retrospective cohort study of patients with severe SARS-CoV-2 infection, combined treatment with corticosteroids and tocilizumab reduced in-hospital mortality by 25%.^[10,11]^ Gamma globulin regulates overreactive immune responses and decreases infection. Antibiotics should be administered to patients with severe SARS-CoV-2 infection to avoid secondary bacterial and/ or fungal infections. In particular, 20% of patients with SARS-CoV-2 infection experience bacterial infection.^[12]^ Other studies have investigated combination therapies for SARS-CoV-2 infection that include anti-inflammatory agents (corticosteroids, tocilizumab, anakinra, and IVIG), convalescent plasma, and remdesivir,^[13]^ which are associated with improved outcomes in hospitalized patients.^[14]^ For patients with advanced cancers, higher-intensity treatment seems necessary.

This study has several limitations. It is retrospective and non-randomized, with a relatively small sample size, and included patients with diverse tumor types, which may have led to heterogeneity in our findings. Future studies with larger sample sizes and prospective study designs are necessary to explore further the risk factors and optimal treatment strategies for this high-risk patient population. Despite these limitations, our study provides valuable insights into the clinical characteristics and treatment outcomes of patients with advanced cancer and SARS-CoV-2 infection.

In conclusion, older age, poor ECOG scores, and the presence of comorbid lung disease are risk factors for severe SARS-CoV-2 infection in patients with advanced cancer. The PROMISING regimen was safe and efficacious in these patients.

## Supplementary information

Table S1. Demographic and baseline clinical characteristics of patients with advanced cancer and SARS-CoV-2 infection.

Table S2. The outcome of different treatments in patients with advanced cancer and SARS-CoV-2 infection.

Table S3. Demographic and baseline clinical characteristics of patients with advanced cancer and treated with different regimens.

Table S4. Treatment-related adverse events associated with the PROMISING regimen and other treatment regimens in patients with advanced cancer and SARS-CoV-2 infection.

Supplementary information of this article can be found online at www.intern-med.com.

## Supplementary Material

Supplementary Material
