# Supplementary material for: Treatment strategies with combined agency against severe viral pneumonia in patients with advanced cancer
Source: J Transl Int Med. 2024 Jul 27;12(3):317–20. doi: 10.2478/jtim-2024-0007 (PMC11285019; doi:10.2478/jtim-2024-0007)
Supplement: Supplementary file 1 — Supplementary Material [file jtim-2024-0007_sm.pdf]

Treatment strategies with combined agency against severe viral pneumonia in patients with advanced cancer

Fan Zhang[1], Tao Li[1], Yibing Bai[1,2], Jiayi Liu[1,3], Jiapei Qin[1,2], An Wang[1,2], Yimin Zhu[4], Min Zhang[5], Zhiqiang Ma[1], Xin Zhou[1,2], Lijie Wang[1], Ming Gao[1], Xiaodong Wu[1], Yan Shao[1], Xiangfei Zhao[6], Juyi Wen[6], Jingzhi Guan[4], Jinliang Wang[1], Junxun Ma[1], Taohai Tao[1], Yi Hu[1]

Supplementary Table S1. Demographic and baseline clinical characteristics of patients with advanced cancer and SARS-CoV-2 infection

**Characteristic**	**Patients (*n* = 89)**			***P*-value**
	All patients (*n* = 89)	Non-severe SARS-CoV-2 (*n* = 33)	Severe SARS-CoV-2 (*n* = 56)	
Age median, years	62 (31-91)	58.5 (31-74)	66 (33-91)	0.005
Gender				
Male	69	23 (33.33%)	46 (66.67%)	0.174
Female	20	10 (50%)	10 (50%)	
Smoking history				
Never smoked	45	22 (48.89%)	23 (51.11%)	0.020
Smoker	44	11 (25%)	33 (75%)	
BMI				
≤ 25	59	23 (38.98%)	36 (61.02%)	0.602
> 25	30	10 (33.33%)	20 (66.67%)	0.602
ECOG				
0-2	57	28 (49.12%)	29 (50.88%)	0.002
3-4	32	5 (15.6%)	27 (84.4%)	
SpO_2				

≤93	31	0 (0.0%)	31 (100.0%)	<0.001
> 93	36	19 (52.78%)	17 (47.22%)	
Distant metastasis	73	27 (36.99%)	46 (63.01%)	0.969
Tumor diagnosis				
Lung cancer	35	11 (31.42%)	24 (68.57%)	0.374
Digestive system cancer	36	14 (38.89%)	22 (61.11%)	0.771
Head and neck cancer	7	3 (42.9%)	4 (57.1%)	1
Genitourinary cancer	10			
Gynecological tumor	5	4 (80.0%)	1 (20.0%)	0.117
Prostate cancer	1	0 (0.0%)	1 (100.0%)	1
Urologic neoplasms	2	0 (0.0%)	2 (100.0%)	0.528
Breast cancer	2	1 (50.0%)	1 (50.0%)	1
Other	1	0 (0.0%)	1 (100.0%)	1
History of prior treatment				
Surgery	32	12 (37.5%)	20 (62.5%)	0.001
Radiotherapy	12	4 (33.33%)	8 (66.67%)	1
Immunotherapy	40	15 (37.5%)	25 (62.5%)	0.941
Chemotherapy	53	24 (45.28%)	29 (54.72%)	0.052
Targeted therapy	24			
Tyrosine kinase inhibitor therapy	10	4 (40.0%)	6 (60.0%)	1
Antiangiogenic therapy	14	4 (28.6%)	10 (71.4%)	0.677
Treatment for cancer <14 d	17	9 (52.94%)	8 (47.06%)	0.132
Checkpoint inhibitor-related pneumonitis				
YES	5	0 (0.0%)	5 (100.0%)	0.137
NO	35	15 (42.86%)	20 (57.14%)	
Symptoms and signs at on admission				

Fever	57	5 (8.77%)	52 (91.23%)	<0.001
Aversion to cold	13	1 (7.69%)	12 (85.7%)	0.039
Cough	49	8 (16.33%)	41 (83.67%)	<0.001
Fatigue	44	4 (9.09%)	40 (90.91%)	<0.001
Dyspnea	46	1 (2.17%)	45 (97.83%)	<0.001
Expectoration	39	2 (5.13%)	37 (94.87%)	<0.001
Chest tightness	48	3 (6.25%)	45 (93.75%)	<0.001
Sore throat	24	5 (20.83%)	19 (79.17%)	0.054
Comorbidity				
Diabetes	13	3 (23.08%)	10 (76.92%)	0.412
Cerebrovascular disease	10	6 (60.0%)	4 (40.0%)	0.213
Hypertension	25	6 (24.0%)	19 (76.0%)	0.11
Comorbid lung disease	43	5 (11.63%)	38 (88.37%)	<0.001
Organ damage indices				
WBC	7.52 (1.64-21.8)	5.95 (1.64-17.44)	7.52 (2.07-21.8)	0.006
Neutrophil count	4.10 (0.80-19.53)	2.74 (0.80-15.8)	5.05 (1.40-19.53)	0.008
IL-6	53.7 (5.08-543.3)	29.59 (5.13-65.31)	63 (5.08-543.3)	0.161
CRP	9.42 (0.18-295.3)	2.193 (0.18-9.4)	13 (0.25-295.3)	0.045
LDH	192.5 (47.1-1695.6)	190.8 (75.9-1695.6)	330.4 (47.1-517.1)	0.012
NLR	4.09 (0.87-82)	2.67 (0.87-28.3)	4.7 (0.93-82)	0.048
Thrombin time	15.1 (10.7-43.2)	15.6 (10.7-18)	14.9 (11.1-43.2)	<0.001
Prothrombin time activity	82.75 (28-130)	105 (46-119)	78 (28-130)	0.002

BMI: Body Mass Index; Eastern Cooperative Oncology Group (ECOG) Performance Status; IL-6: Interleukin-6; CRP: C-reactive protein; LDH: Lactic dehydrogenase; PLT: platelet.

Supplementary Table S2. The outcome of different treatments in patients with advanced cancer and SARS-CoV-2 infection

	**Cured**	**Deaths**	**Total**	***P*-value**
Total	**39**	17	56	0.049
PROMISING regimen	7	0	7	
Five-medication regimens	2	1	3	
Four-medication regimens	4	2	6	
Three-medication regimens	13	6	19	
Two-medication regimens	13	8	21	

Supplementary Table S3. Demographic and baseline clinical characteristics of patients with advanced cancer and treated with different regimens

**Characteristic**	**Patients**			***P*-value**
	**All patients (*n* = 28)**	**PROMISING regimen (*n* = 7)**	**Other regimens (*n* = 21)**	
Age median, years	60 (38-79)	58.5 (49-76)	62 (38-79)	
Sex				1
Male	14 (50)	4	10	
Female	14 (50)	3	11	
Smoking history				1
Never smoked	14 (50)	4	10	
Smokers	14 (50)	3	11	
BMI				1
≤ 25	17 (60.7)	4	13	
> 25	11 (39.3)	3	8	
ECOG				
0-2	6 (21.43)	1	5	0.595
3-4	22 (78.57)	6	16	
SpO_2				0.290
≤93	23 (82.1)	7	16	
>93	5 (17.9)	0	5	
Tumor diagnosis				0.747
Lung cancer	8	3	5	
Digestive system cancer	7	2	5	
Genitourinary cancer	6	1	5	

Breast cancer	4	1	3	
Other	3	0	3	
Comorbid lung disease				0.198
YES	13(46.4)	5	8	
NO	15(53.6)	2	13	

Supplementary Table S4. Treatment-related adverse events associated with the PROMISING regimen and other treatment regimens in patients with advanced cancer and SARS-CoV-2 infection

	**All patients (*n* = 56)**	**PROMISING regimen Group (*n* = 7)**	**Other regimens group (*n* = 49)**
Secondary infection	17	0	17
Hyperglycemia	14	3	11
Dysgustatory	1	0	1
Hypertension	2	1	1
Myelosuppression	2	0	1
Renal damage	5	0	5
Liver damage	6	2	4
Gastrointestinal reaction	11	1	10
Peripheral edema and hypersensitivity	1	0	1
Ecchymosis	1	0	1
Gingival bleeding	2	0	2
Hematuria	2	0	2
